# *Fusobacterium nucleatum* and oral cancer: a critical review

**DOI:** 10.1186/s12885-021-08903-4

**Published:** 2021-11-13

**Authors:** Emily McIlvanna, Gerard J. Linden, Stephanie G. Craig, Fionnuala T. Lundy, Jacqueline A. James

**Affiliations:** 1 Patrick G Johnson Centre for Cancer Research, Queen’s University Belfast, Belfast, Northern Ireland, UK; 2 Centre for Public Health, School of Medicine, Dentistry and Biomedical Sciences, Queen’s University Belfast, Belfast, Northern Ireland, UK; 3 Precision Medicine Centre of Excellence, Health Sciences Building, Queen’s University Belfast, Belfast, Northern Ireland, UK; 4 Wellcome-Wolfson Institute for Experimental Medicine, Queen’s University Belfast, Belfast, Northern Ireland, UK; 5 Northern Ireland Biobank, Health Sciences Building, Queen’s University Belfast, Belfast, Northern Ireland, UK

**Keywords:** Fusobacteria, *F. nucleatum*, Carcinogenesis, Oral cancer, OSCC

## Abstract

There is a growing level of interest in the potential role inflammation has on the initiation and progression of malignancy. Notable examples include *Helicobacter pylori*-mediated inflammation in gastric cancer and more recently *Fusobacterium nucleatum*-mediated inflammation in colorectal cancer. *Fusobacterium nucleatum* is a Gram-negative anaerobic bacterium that was first isolated from the oral cavity and identified as a periodontal pathogen. Biofilms on oral squamous cell carcinomas are enriched with anaerobic periodontal pathogens, including *F. nucleatum,* which has prompted hypotheses that this bacterium could contribute to oral cancer development. Recent studies have demonstrated that *F. nucleatum* can promote cancer by several mechanisms; activation of cell proliferation, promotion of cellular invasion, induction of chronic inflammation and immune evasion. This review provides an update on the association between *F. nucleatum* and oral carcinogenesis, and provides insights into the possible mechanisms underlying it.

## Background

Head and neck cancer was identified as the seventh most common cancer worldwide in 2018, with 890,000 new cases and 450,000 deaths being reported [1]. Oral squamous cell carcinoma (OSCC) is the most prevalent subgroup of head and neck cancer and represents a major cause of morbidity and mortality worldwide. OSCC has a remarkably high prevalence in some populations, particularly in Southern Asia and the Pacific islands, and is a leading cause of cancer death among men in India and Sri Lanka [1]. Some OSCCs arise from oral potentially malignant disorders (OPMDs) such as leukoplakia and erythroplakia, whilst others arise de novo [2]. Established risk factors for OSCC include smoking and oral exposure to tobacco, which in South Asia may be associated with habitual use of betel quid. The consumption of alcohol is a recognised risk factor and has a synergistic interaction with smoking [3]. Although OSCC predominantly affects males in their sixth or seventh decade, the incidence of OSCC in women and in people under 40 is increasing [4]. Moreover, emerging evidence suggests that a subgroup of those presenting with OSCC have never smoked or drank alcohol [5]. This implies that there are other unknown aetiological factors that are associated with the development of OSCC.

In recent years it has been shown that specific infectious agents play a key role in the development of certain cancer types [6]. In the context of head and neck cancer, human papilloma virus (HPV) type 16 has been identified as a causative agent for oropharyngeal cancer [7]. HPV-induced oropharyngeal tumours are considered a separate disease entity which have a better prognosis compared to HPV-ve tumours [7]. The favourable outcome of HPV + ve compared with HPV-ve oropharyngeal tumours is so substantial that the tumour-node-metastasis (TNM) staging for HNSCC was adapted in the eighth edition to include p16 immunostaining as a surrogate marker for HPV status [7]. The percentage of head and neck cancers diagnosed as HPV-positive oropharyngeal cancers in the United States rose from 16.3% in the 1980s to more than 72.7% in the 2000s [8]. The oral mucosa is exposed to a huge array of microorganisms that collectively comprise the oral microbiome. Studies using both traditional culture and culture independent molecular approaches have identified around 700 predominant bacterial species in the oral cavity [9]. The presence of several distinct habitats, including the hard non-shedding tooth surfaces in the oral cavity, presents unique microbial niches that can communicate oral microbiome changes at micron-scale gradients with each other via saliva for both short and long range microbial interactions [9]. The accumulation and maturation of dental plaque on tooth surfaces can lead to gingivitis, which is reversed on plaque removal [10]. In some cases, persistent accumulation of dental plaque biofilms and associated chronic inflammation causes periodontitis, resulting in irreversible destruction of tooth supporting tissues [10]. In recent years there has been a reappraisal of our understanding of the bacterial pathogenesis of periodontitis. It is now accepted that shifts in the microbiome induced by inflammation favour overgrowth of certain commensals and altered expression of virulence factors, rather than the introduction of new pathogenic species [11, 12]. Inflammation has long been suspected to play a major role in the pathogenesis of cancer, and it has been hypothesised that commensal microorganisms might provide the link between chronic inflammation and carcinogenesis [13]. Interestingly, several studies have identified periodontitis as an independent risk factor for oral cancer development [14–17]. One microorganism that is a key player in the development and maturation of biofilms that accompany dysbiotic changes in dental plaque is *Fusobacterium nucleatum* [10].

Landmark publications in 2012, from two independent groups, reported that *F. nucleatum* infection was prevalent in human colorectal carcinoma (CRC) [18, 19]. Subsequently there has been extensive research into *F. nucleatum* as a risk factor for CRC however, its putative involvement in oral cancer has received less attention. This review will focus on a possible role for *F. nucleatum* in oral cancer with discussion of possible mechanisms that this bacterium could utilise to promote neoplastic change in the oral mucosa. It will also identify questions raised by the potential involvement of this common constituent of the oral microbial flora in oral carcinogenesis.

### *Fusobacterium nucleatum*

*F. nucleatum* is a Gram-negative filamentous spindle-shaped rod that is a common inhabitant of the oral flora. It has not traditionally been considered as a pathogenic species in the oral cavity, although it has an emerging role in driving inflammation. There is speculation that it can act as an opportunist pathogen in relation to extra-oral sites, as it has been implicated in diseases such as appendicitis, brain abscesses, and chorioamnionitis [20]. However further discussion of this, except for involvement in CRC, is beyond the scope of this review. *F. nucleatum* is the second most frequently recovered species in dental plaque biofilms associated with health [12]. *F. nucleatum* is termed a core species in oral biofilms as its proportions remain unchanged, at about 25%, in both health- and disease-associated dental plaque. However, this should be interpreted in the context of a 3-log increase in the total microbial load that occurs in periodontal inflammation [12]. *F. nucleatum* is a pivotal ‘bridging’ bacterium that acts in a supportive role by co-aggregating with both the early (Streptococcal spp.) and late colonizers, such as *Porphyromonas gingivalis,* thereby guiding the architecture of the dental plaque biofilm [21]. Its long rod shape is central to establishing structural relationships that are critical to polymicrobial biofilms and interactions between microorganisms [20]. *F. nucleatum* can bind and/or invade diverse cell types including oral, colonic and placental epithelial cells, T-cells, keratinocytes and macrophages through the expression of adhesins such as FadA and Fap2 [22, 23]. These adhesins are also thought to have a putative role in carcinogenesis [20].

### *F. nucleatum* in gastrointestinal cancer

Many studies have shown an enrichment of *F. nucleatum* in CRC compared to the levels in normal adjacent tissue and in healthy controls [19, 24–28]. A recent meta-analysis indicated that the odds of *F. nucleatum* DNA being detected were higher in colorectal tumour tissue compared with adjacent healthy tissue and healthy tissue from controls [29]. *F. nucleatum* DNA was also higher in colorectal polyp tissue compared with healthy tissue from controls [29]. Studies have shown that *F. nucleatum* is abundant in faecal samples from patients with CRC [30–32]. Meta-analysis found the pooled odds of *F. nucleatum* positivity were higher in faecal samples from patients with CRC compared with healthy controls; higher in patients with CRC compared with individuals with colorectal polyps; but not from individuals with colorectal polyps compared with healthy controls [29]. *F. nucleatum* has been isolated from cancers at other sites along the digestive tract, namely the pancreas [33], oesophagus [34, 35] and stomach [35, 36]. *F. nucleatum* has also recently been implicated in the growth and progression of breast cancer [37].

*F. nucleatum* was previously regarded as a passive bacterium in the gastrointestinal tract. However, it is now recognised that *F. nucleatum* infection can induce a series of specific tumour molecular events in colorectal cancer, including CpG island methylator phenotype, microsatellite instability and genetic mutations in *BRAF* and *TP53* [24, 26, 38]. Moreover, many of these studies have identified that the presence of intra-tumoral *F. nucleatum* is associated with worse survival [26–28, 33, 35, 38–46]. It has also been observed that *F. nucleatum* infection is associated with worse clinicopathological features such as larger tumours, poorer differentiation, lymph node and distant metastases, advanced tumour stage and deeper tumour invasion [19, 24, 27, 40–42].

Interestingly, it has been shown that strains of *F. nucleatum* in CRC were identical to strains of this species isolated from the mouth, suggesting that the intra-tumoral *F. nucleatum* may have originated from the oral cavity [47]. If *F. nucleatum* from the oral cavity has a role in cancer development at extra-oral sites, then it is reasonable to hypothesise that this bacterium could contribute to carcinogenesis in the oral cavity itself.

### *F. nucleatum* in OSCC

Several studies aimed to identify the microbial species present within OSCC tumour tissue compared with non-tumorous control materials using either culture approaches, 16 s rRNA sequencing or next generation sequencing (NGS), and these studies have already been previously reviewed [48–52]. In 1998, the first association study by Nagy et al. found that levels of *Porphyromonas* and *Fusobacterium* were significantly higher in OSCC than in normal tissue [53]. However, more recent studies have profiled tumour-specific microbiomes at the species level using NGS which has facilitated the detection of *F. nucleatum* in oral cancer samples [54–62].

Using NGS, Al-Hebshi and colleagues found that *F. nucleatum* was the most abundant species in OSCC samples, followed by *Pseudomonas aeruginosa* [55]. This study was also the first to report on the potential functional role of the OSCC-associated bacteriome as it found that genes involved in bacterial mobility, flagellar assembly, bacterial chemotaxis and lipopolysaccharide (LPS) synthesis were enriched in the tumours [55]. The latter being particularly relevant to the virulence of Gram-negative bacteria, such as *F. nucleatum*. Recently, Zhang et al. confirmed that the abundance of *F. nucleatum* was significantly increased in OSCC [56]. Furthermore, this study corroborated the finding that the abundance of genes involved in bacterial chemotaxis, flagellar assembly and importantly, LPS biosynthesis, were significantly increased in the OSCC group [56]. Similarly, Zhao and colleagues identified *F. nucleatum* to be one of three *Fusobacterium* species significantly enriched in the oral cancer group, whereas *P. gingivalis* did not differ in abundance between groups [57]. Additionally, several operational taxonomic units associated with *Fusobacterium* were highly involved in OSCC and demonstrated good diagnostic power [57]. Perera and colleagues identified enrichment of the LPS biosynthesis pathway in OSCC tissue and speculated that the ‘*Fusobacterium* oral taxon 204’ detected in their study may have been a functional equivalent to *F. nucleatum* [59]. Yost and colleagues profiled RNA expression in the oral microbiome in OSCC and reported that Fusobacteria had a higher number of transcripts at tumour sites compared with adjacent non-affected sites or healthy controls. Specifically, *F. nucleatum* showed the highest upregulation of putative virulence factors for tumour sites. They concluded that Fusobacteria was the phylogenetic group responsible for the upregulation of virulence factors in the oral microbiome of OSCC patients [62].

To date, only one systematic review and meta-analysis on the presence of *Fusobacterium* in oral cancer/head and neck cancer has been completed [63]. This study concluded that *Fusobacterium* is present and in higher abundance in oral cancer/head and neck cancer samples when compared to non-cancer samples, suggesting that *Fusobacterium* could contribute to oral cancer/head and neck cancer development [63]. However, it is also possible that tumour colonisation by *F. nucleatum* reflects its ability to exploit and replicate effectively in the hypoxic tumour microenvironment. Perhaps dispelling this hypothesis is the finding that OPMDs are also enriched with *F. nucleatum* [64, 65]. This evidence that *F. nucleatum* colonisation begins early in the process of malignant transformation supports a potential role for microbiome changes in the pathogenesis of the disease.

Two recently published studies have examined the prognostic effect of *F. nucleatum* in oral/head and neck cancer, and the findings are summarised in Table 1 [66, 67]. Neuzillet et al found that *F. nucleatum* was significantly associated with improved overall survival, relapse-free survival and metastasis-free survival in their merged OSCC cohort [66]. Similarly Chen et al found that *F. nucleatum* enrichment in HNSCC tumour tissues was significantly associated with better cancer-specific survival and a lower rate of relapse [67]. These findings are unexpected given its association with poor prognosis in other cancer types. *F. nucleatum*-associated OSCC was more frequent in HPV-ve tumours and in older patients lacking the traditional risk factors of alcohol [66] and smoking [67]. *F. nucleatum*-positivity was also associated with lower tumour (pT) stage [67] and lower nodal (pN) stage [66]. Interestingly, the association of low pT or pN stage with *F. nucleatum* positivity allowed the identification of a patient subgroup with remarkably good prognosis [66].

**Table 1:** Summary of publications reporting on the prognostic impact of *F. nucleatum* in oral cancers

Author	Reference	Specimen type	Detection method	Number of cases	***F. nucleatum*** detection rate	Prognostic impact of ***F. nucleatum*** detection	Molecular and clinicopathological associations with tumour ***F. nucleatum*** positivity
Neuzillet et al (2021)	66	Fresh frozen OSCC	qPCR	151	82.1% *Fn*-positive (124/151)	Better OS, RFS and MFS	Older (> 56 years), non-drinkers, low pN stage. Low RNA levels of M2 macrophages (CD163), CD4 lymphocytes, fibroblasts (PDGFRß), TLR4, OX40 ligand (TNFSF4) High levels of TNFSF9 and IL-1ß
Chen et al (2020)	67	Fresh frozen HNSCC	qPCR	68	55.8% *Fn*-high (38/68)	Better CSS and RFS	Non-smokers, lower tumour stage, hypermethylation of *LXN* and *SMARCA2* genes

Abbreviations: CIMP-H, CpG island methylator phenotype high; CSS, cancer-specific survival; ddPCR, droplet digital polymerase chain reaction; DFS, disease-free survival; FFPE, formalin-fixed paraffin-embedded; MSI-H, microsatellite instability-high; OS, overall survival; qPCR, quantitative polymerase chain reaction; RFS, recurrence-free survival; RT-qPCR, real time quantitative polymerase chain reaction

### Potential carcinogenesis mechanisms linked to *F. nucleatum*

**Fig. 1:**
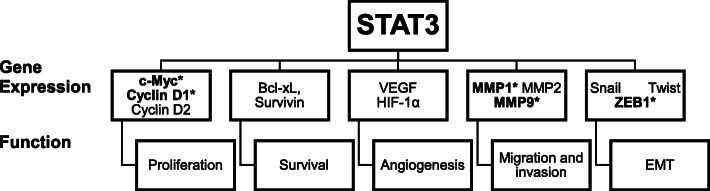
Oncogenic role of STAT3. STAT3 controls various tumour-associated genes which can influence proliferation, angiogenesis, invasion and metastasis. *Genes (in bold) known to be upregulated as a result of STAT3 activation in oral cancer cells infected with *F. nucleatum*

Until recently, the only experimental evidence that *F. nucleatum* could induce malignant change in the oral cavity was presented by Binder Gallimindi et al. who showed that *P. gingivalis* and *F. nucleatum* could promote carcinogenesis in a chemically-induced murine model of OSCC [68]. Both Gram-negative anaerobic pathogens could stimulate tumorigenesis via direct interaction with oral epithelial cells through Toll-like receptors (TLR) and augmented signalling via the IL-6-STAT3 axis [68]. Infection with *F. nucleatum* induced key molecular players, such as cyclin D1 and matrix metalloproteinase-9 (MMP-9), which are involved in oral tumour growth and invasiveness. Tumours from infected mice were 2.5 times larger and were significantly more invasive compared to non-infected mice [68]. A more recent study by Harrandah et al. supported these findings in a similar oral tumour murine model [69]. Infected oral cancer cells had upregulated expression levels of MMP1, MMP9, and IL-8. The expression of cell survival markers MYC, JAK1, and STAT3 and epithelial-mesenchymal transition markers ZEB1 and TGF-β were also significantly elevated [69]. Additionally, mice infected with *F. nucleatum* developed significantly larger and more numerous lesions compared to uninfected controls [69]. Both studies identified the signal transducer and activator of transcription-3 (STAT3) signalling pathway as being a key mediator in fostering oral tumorigenesis. STAT3 signalling promotes initiation and progression of cancer by controlling genes responsible for suppressing apoptosis and driving proliferation, angiogenesis, metastasis and invasion (Fig. 1) [68].

Most of the research on mechanisms linking *F. nucleatum* with carcinogenesis has focused on how *F. nucleatum* contributes to CRC, therefore conclusions relating to its mechanistic involvement in oral carcinogenesis are tentative. However, the mechanisms that have been identified through which *F. nucleatum* promotes neoplastic change in the colon could be applicable at other mucosal sites and so they merit discussion in the context of OSCC.

#### Binding and localization

Recent evidence has shown that *F. nucleatum* attaches via its Fap2 adhesin, to an oligosaccharide called Gal-GalNAc that is overexpressed on cancer cells [37, 70]. Expression of Gal-GalNAc can be detected using the lectin Peanut Agglutinin (PNA) which is specific for this oligosaccharide [71]. It has been shown that PNA staining, and thus Gal-GalNAc levels, correlates with human breast cancer progression. Furthermore, the occurrence of *F. nucleatum* gDNA in breast cancer samples correlates with high Gal-GalNAc levels [37]. Fap2-dependent binding of *F. nucleatum* to breast and colon tumours has been demonstrated [37, 70]. No studies to date have investigated if *F. nucleatum* colonisation of OSCC tissue occurs via a Fap2/Gal-GalNAc dependent mechanism. However, PNA has been shown to preferentially bind to OSCC tissue, which is indicative of Gal-GalNAc expression. Furthermore, one study found that PNA showed weak binding to normal oral mucosal cells, intermediate binding to dysplastic cells and strong binding to malignant squamous cell carcinoma [72]. This coincides with the finding that *F. nucleatum* abundance increases along the pathway from normal tissue to dysplasia to malignancy [64, 65].

#### Co-aggregation with other microorganisms

*F. nucleatum* is a key ‘bridging’ organism between early and late colonisers and its outer membrane adhesin Fap2 is partly responsible for facilitating multispecies biofilm formation [23] *F. nucleatum* is always present when *P. gingivalis* is reported within oral biofilms, suggesting that it precedes this species and is required for its colonization [21]. *P. gingivalis* is an acid-sensitive bacterium, however *F. nucleatum* can produce ammonia by fermenting glutamate and aspartate in order to provide a more neutral environment conducive for *P. gingivalis* colonisation [73]. A study by Katz et al. was the first to identify *P. gingivalis* in OSCC tissue [74]. Further studies have suggested that *P. gingivalis* could also contribute to OSCC and digestive tract cancer development [75]. It is possible that *F. nucleatum* and *P. gingivalis* work co-operatively to promote neoplastic changes by triggering chronic inflammation. Indeed, Binder Gallimindi et al. showed that a mixture of *F. nucleatum* and *P. gingivalis* significantly stimulated in vitro proliferation of human OSCC cells [68]. However, a more recent study by Harrandah et al. showed that infection of oral cancer cells with *F. nucleatum* alone had comparable or greater effects than a combination of four periodontal pathogens [69].

*Candida albicans* is an opportunistic pathogenic yeast that is commonly found in the gastrointestinal tract and mouth [76]. Recent mycobiome studies have shown increased abundance of several fungal species including *C. albicans* in OSCC [76]. It is well established that *Candida* species in the oral cavity possess the enzyme alcohol dehydrogenase responsible for catalysing the production of acetaldehyde, a potent carcinogen, from alcohol [48]. It has been shown that *F. nucleatum* co-aggregates with *Candida* species and this can facilitate colonisation [77]. Amer et al. reported that *Candida* colonisation of oral leukoplakia was associated with increased levels of *F. nucleatum* [64]. It is possible that *F. nucleatum* may indirectly act to increase oral cancer risk by increasing the exposure of oral mucosa to acetaldehyde produced by *Candida* species.

#### Activation of cell proliferation

Cancer is, at its simplest, uncontrolled cell growth, and *F. nucleatum* has been shown to influence the proliferation of cancer cells by interacting with endothelial cadherin (E-cadherin) [78, 79]. Fusobacterial FadA binds to E-cadherin which is expressed on the surface of the host cell membrane [80]. E-cadherin is a tumour suppressor which acts through β-catenin. Interaction of fusobacterial FadA with E-cadherin activates WNT/β-catenin signalling, resulting in cell proliferation with increased expression of oncogenic and inflammatory genes [78, 81].

Similarly, *F. nucleatum* has been shown to increase CRC proliferation in a mouse xenograft by activating Toll-Like Receptor 4 (TLR4) signalling to MYD88, leading to activation of the nuclear factor NFκB and increased expression of miR21; this miRNA reduces levels of the RAS GTPase RASA1 which is responsible for controlling cell proliferation and differentiation [82].

Cell cycle progression is facilitated by cyclin-dependent kinases that are activated by cyclins. *F. nucleatum* infection has been associated with the activation of cyclin D1, which facilitates intestinal tumorigenesis [81, 83]. In OSCC, both *F. nucleatum* and *P. gingivalis* were shown to be capable of significantly stimulating OSCC cell proliferation by upregulating cyclin D1 and c-Myc [68, 69]. Bacterial activation of TLR4 led to an increased expression of interleukin-6 (IL-6) which in turn activated STAT3, a key signalling molecule responsible for regulating cyclin D1 and c-Myc [68, 69]. Moreover, *F. nucleatum* was shown to cause DNA damage and promote cell proliferation in oral cancer cells by decreasing p27 expression, a cyclin-dependent kinase inhibitor, and accelerating the cell cycle [84]. Additionally, *F. nucleatum* downregulated the DNA repair proteins Ku70 and p53, thereby weakening cell repair ability [84].

A recent study identified that enrichment of *F. nucleatum* in HNSCC was associated with host gene promoter methylation, including hypermethylation of tumour suppressor genes *LXN* and *SMARCA2* [67]. *SMARCA2* is a gene involved in ATP-dependent chromatin remodelling related to DNA repair and replication. This suggests that *F. nucleatum* infection may cause cell proliferation through epigenetic silencing [67].

#### Induction of inflammation

The pro-inflammatory potential of *F. nucleatum* is well documented, as it is known to facilitate reactive oxygen species (ROS) generation and cytokine production [85–89]. Chronic inflammation plays a pivotal role in carcinogenesis and may explain the strong association between periodontitis and higher risk of OSCC [48]. *F. nucleatum* has been found to be associated with high cytokine levels in CRC and OSCC, creating an inflammatory microenvironment supportive of tumour progression [18, 68, 69]. LPS, which is found in the outer membrane of *F. nucleatum*, activates the TLR4-mediated NF-κB signalling pathway to produce pro-inflammatory cytokines such as IL-6, IL-8 and tumour necrosis factor alpha [68].

#### Anti-tumour immune response

*F. nucleatum* has been shown to recruit myeloid-derived suppressor cells into the tumour microenvironment in the ApcMin/+ mouse model [18]. Myeloid-derived suppressor cells can inhibit T-cell proliferation and induce T-cell apoptosis [18]. This is consistent with a recent finding of the inverse association between the amount of *F. nucleatum* and the density of CD3 and CD4 T-cells in colorectal and breast cancer tissue [37, 90, 91]. A significant negative association between *F. nucleatum* load in OSCC and markers of B lymphocytes, CD4 T helper lymphocytes, M2 macrophages and fibroblasts has also been observed [66]. *F. nucleatum* inhibitory protein can also inhibit human T-cell activation by arresting cells in the G1 phase of the cell cycle [92]. The Fusobacterial Fap2 adhesin binds and activates the T-cell immunoreceptor with Ig and ITIM domains (TIGIT), which is an immunoregulatory signalling receptor in T-cells and natural killer (NK) cells [93]. This Fap2-TIGIT interaction protects both *F. nucleatum* and nearby tumour cells from being killed by immune cells [93]. Local immune suppression can also occur because Fap2 and RadD outer membrane proteins of *F. nucleatum* induce cell death in lymphocytes [94]. *F. nucleatum* also exerts an immunosuppressive effect by promoting M2 polarization of macrophages in *F. nucleatum*-related CRCs, possibly through the TLR4/IL-6/p-STAT3/c-MYC signalling pathway [95].

#### Cell migration and invasion

Matrix metalloproteinases (MMPs) are a family of zinc-dependent endopeptidases collectively capable of degrading all components of the extracellular matrix (ECM) [96]. MMPs play a role in pathological conditions with excessive degradation of ECM, including tumour invasion and metastasis [96]. Both *P. gingivalis* and *F. nucleatum* can produce MMPs via different mechanisms and so promote cancer cell invasion and metastasis [97–100]. In OSCC, it has been observed that exposure to *P. gingivalis* and *F. nucleatum* resulted in the induction of MMP-1 and MMP-9 [68, 69]. Similarly, AT3 mouse mammary carcinoma cells incubated with *F. nucleatum* also exhibited an overexpression of MMP-9 [37].

Epithelial-mesenchymal transition (EMT), is defined as the process by which epithelial cells adopt a mesenchymal phenotype and is a phenomenon observed in cancer development and progression [101]. In general, cells proceeding to EMT exhibit down-regulation of epithelial markers such as E-cadherin and up-regulation of mesenchymal markers, including neural-cadherin (N-cadherin) and Vimentin [102]. This switch in cell differentiation behaviour is controlled by a group of transcription factors including the zinc-finger E-box-binding homeobox 1 and 2 proteins (ZEB1/2), SNAIL and TWIST. High levels of *F. nucleatum* in CRC are negatively correlated with E-cadherin expression but positively correlated with expression of N-cadherin [45]. Similarly, exposure of OSCC cell lines to *F. nucleatum* has been associated with a significant decrease in transcription of E-cadherin and the upregulation of N-cadherin, vimentin and Snail [103, 104].. *F. nucleatum* can upregulate the expression of ZEB1 in oral cancer cells to induce this mesenchymal state, [66] a mechanism which has previously been identified in *H. pylori*-infected gastric epithelial cells [105].

### Possible implications for OSCC management

Since the discovery that *F. nucleatum* is an important biomarker for CRC, particularly a prognostic one, there has been considerable research surrounding potential therapeutic and prevention strategies to address the association of *F. nucleatum* with tumorigenesis. A recent study showed that treatment of mice bearing a colon cancer xenograft with the antibiotic metronidazole successfully decreased *Fusobacterium* load, cancer cell proliferation and tumour growth [46]. Similarly, in an AT3 orthotropic mammary cancer model, metronidazole prevented tumour enlargement and lung metastasis in mice inoculated with *F. nucleatum* [37]. However, antibiotic administration is associated with issues including generation of resistant strains, misbalancing the resident body flora and inducing hypersensitivity reactions. It is possible that some of these issues could be mitigated by using topical metronidazole in the oral cavity for *F. nucleatum*-positive OSCC. Oral rinses could potentially be used as non-invasive samples to reflect tissue microbial composition for diagnostics, as a recent study noted similar relative abundances of bacteria across both oral cancer tissue samples and oral rinses obtained from the same patients [67].

A recent study investigating the role of *Treponema denticola* in promoting oral cancer development showed that the three periodontal pathogens (*T. denticola*, *P. gingivalis* and *F. nucleatum*) enhanced OSCC cell migration, invasion, tumorsphere formation, and tumorigenesis in vivo and that Nisin inhibited these pathogen*-*mediated processes [106]. Nisin is a bacteriocin produced by Gram-positive *Lactococcus* and *Streptococcus* species and is a commonly used food preservative. Nisin has been previously shown to attenuate oral tumorigenesis and thus has therapeutic potential as an antimicrobial and anti-tumorigenic agent [106, 107].

Given the global health burden of colorectal cancer, and other conditions that have been associated with *F. nucleatum,* the development of a vaccine warrants consideration [20]. A vaccine targeting FomA, an outer membrane protein of *F. nucleatum* responsible for bacterial co-aggregation and biofilm formation, has been tested as an agent to combat periodontal infection and halitosis [108]. However, it is not known whether recipients of this vaccine had a lower incidence of cancer attributable to *F. nucleatum* infection. A recent study investigating immunization with the alkyl hydroperoxide reductase subunit C from *F. nucleatum* found that vaccination lowered the levels of the bacterium in intestinal tissues and elicited IgA and IgG responses in mice [109]. Furthermore, clinical isolates of fusobacterial strains naturally lacking Fap2 or inactivated Fap2 mutants, showed reduced binding to Gal-GalNAc on colorectal and breast cancer cells [37, 70]. Therefore, vaccines targeting Fusobacterial Fap2 could theoretically reduce fusobacteria colonisation and potentiation of oral cancer.

## Conclusions

Despite a wealth of research on *F. nucleatum* over several years, many unanswered questions remain. One key area of controversy is whether *F. nucleatum* is an active conductor of neoplastic change in epithelial cells or a passive passenger that colonises due to favourable conditions provided by the tumour milieu. *F. nucleatum* has been primarily characterised as a bridging organism in the assembly and architecture of multi species biofilms however, more recent studies have identified other active roles. The potential for *F. nucleatum* to act as a carcinogen is credible, as it has been shown to promote inflammation and suppress local immune responses. One intriguing question is why a microorganism that is ubiquitous in the mouth throughout life might only very occasionally become carcinogenic? The answer to this likely involves changes within the oral microbiome within the context of host factors such as genetics, oral hygiene behaviour, nutrition, age and exposure to risk factors such as tobacco and alcohol. A recent study found that tobacco, irrespective of the mode of use, created an oral microenvironment favouring anaerobes such as *Fusobacterium* [110].

A “two-hit” model in carcinogenesis, with somatic mutations serving as the first hit and *F. nucleatum* as the second hit exacerbating cancer progression after benign cells become cancerous, has previously been proposed [80]. Inflammatory cytokines and reactive oxygen species produced as a result of *F. nucleatum* infection could facilitate cancer development by inducing mutations, genomic instability and epigenetic alterations [48]. Cytokines can then activate key transcription factors such as NF-κB and STAT3 within oral pre-malignant cells which subsequently promote pro-malignant processes such as proliferation, invasion and metastasis.

**Fig. 2:**
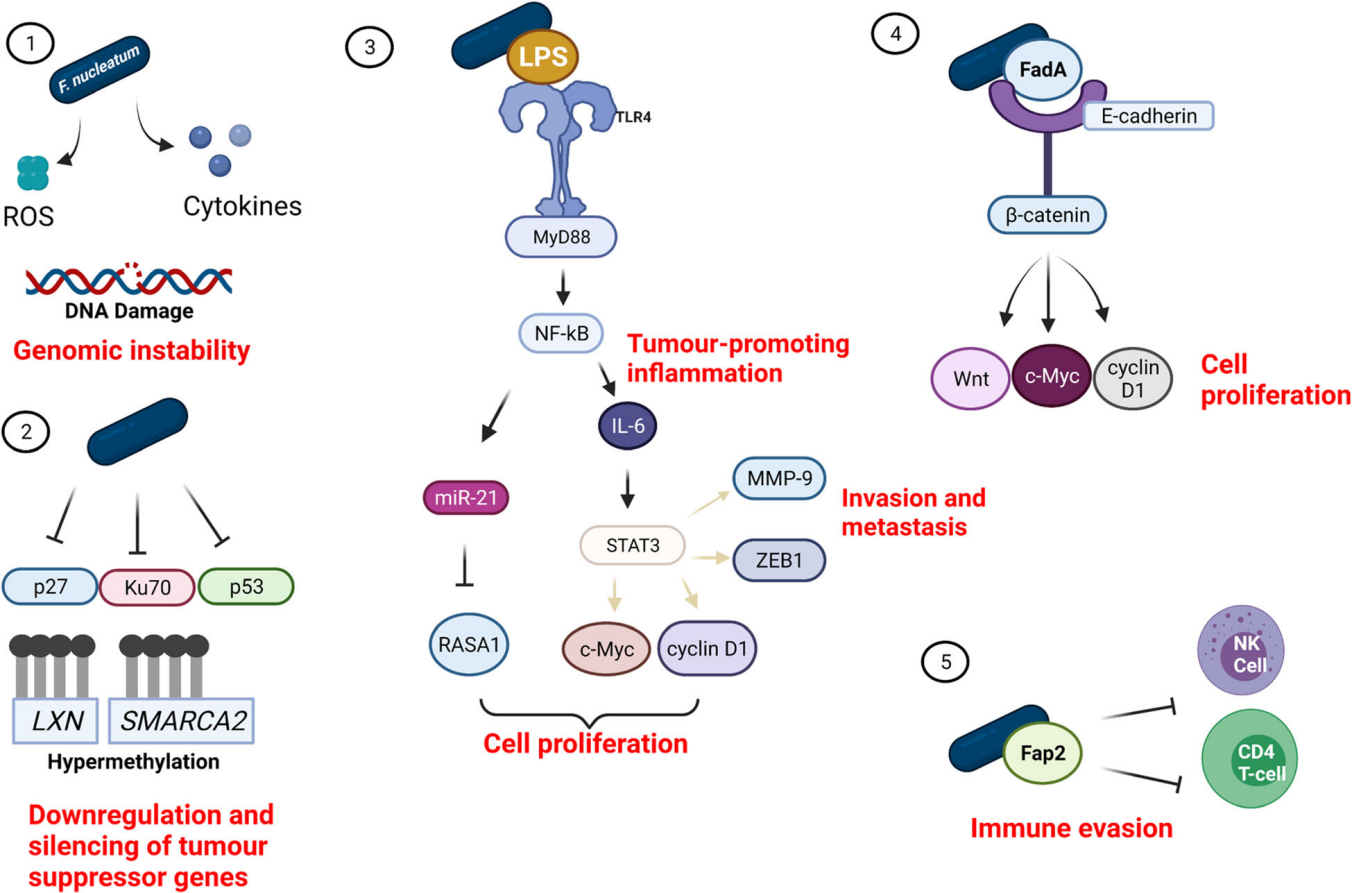
Hallmarks of cancer influenced my *F. nucleatum* infection. (1) Production of reactive oxygen species (ROS) and cytokines by *F. nucleatum* causes DNA damage resulting in genomic instability. (2) *F. nucleatum* infection in HNSCC causes hypermethylation of CpG islands located in the promoter regions of tumour suppressor genes *LXN* and *SMARCA2* resulting in their inactivation. Downregulation of p27, Ku70 and p53 tumour suppressor genes in OSCC results in weakened cell repair ability and increased cell proliferation. (3) LPS/TLR4 signalling results in cytokine production and NF-κB activation which is responsible for tumour-promoting inflammation. Activation of STAT3 upregulates multiple genes responsible for cell proliferation, invasion and metastasis. Upregulated expression of microRNA-21 promotes proliferation of cancer cells. (4) Fusobacterial FadA binds to E-cadherin resulting in decreased phosphorylation of β-catenin. Subsequently, β-catenin translocates to the nucleus, resulting in cell proliferation with increased expression of oncogenic and inflammatory genes. (5) Fusobacterial Fap2 can protect tumours from immune cell attack by inhibiting T-cells and Natural Killer cells. Figure created with BioRender.com

*F. nucleatum* affects many of the accepted hallmarks of cancer [110]. *F. nucleatum* infection can induce genomic instability by causing DNA damage; sustain proliferative signalling via LPS/TLR4 and FadA/E-cadherin signalling pathways; downregulate and silence tumour suppressor genes; avoid immune destruction by inhibiting T-cell and NK cell activities; generate pro-tumour inflammation by activating NF-κB signalling; and cause invasion and metastasis by inducing EMT.

(Fig. 2) [111]. However, further studies are required to fully understand the unique molecular and cellular pathogenic mechanisms of *F. nucleatum* in OSCC tumorigenesis. Although studies have shown that OPMDs are enriched with Fusobacteria [64, 65], direct evidence that this colonisation increases the risk of malignant transformation is absent. There is a need for longitudinal follow-up studies of OPMDs to establish if those enriched with *F. nucleatum* are at an increased risk of developing OSCC independent of smoking, alcohol and HPV status. Advancement of omics technologies could facilitate novel insights in this area. Moreover, further research is necessary to confirm recent findings that *F. nucleatum* infection is associated with a better clinical outcome in OSCC. Validation of *F. nucleatum* as a prognostic biomarker could have major implications for future oral cancer screening and management.

## Data Availability

Not applicable.

## Abbreviations

CRC: colorectal cancer
EMT: epithelial-mesenchymal transition
*F. nucleatum*: *Fusobacterium nucleatum*
FadA: Fusobacterium adhesin A
Fap2: fibroblast activation protein 2
Gal-GalNAc: galactose/N-acetylgalactosamine
HPV: human papilloma virus
IL: interleukin
JAK: Janus Kinase
LPS: lipopolysaccharide
MMP: matrix metalloproteinase
MYC: myelocytomatosis
MYD88: Myeloid differentiation primary response 88
NF-κB: nuclear factor kappa-light-chain-enhancer of activated B cells
NGS: next generation sequencing
NK cell: Natural killer cell
OPMD: oral potentially malignant disorder
OSCC: oral squamous cell carcinomaPNA peanut agglutinin
ROS: reactive oxygen species
STAT: signal transducer and activator of transcription
TIGIT: T cell immunoreceptor with Ig and ITIM domains
TLR: Toll-like receptor
TNF: tumour necrosis factor
WNT: Wingless-type MMTV integration site family member
ZEB: Zinc finger E-box-binding homeobox
